# Evaluation of the occurrence of work‐related musculoskeletal pain among anesthesiology, intensive care, and surgical nurses: An observational and descriptive study

**DOI:** 10.1111/nhs.12767

**Published:** 2020-09-03

**Authors:** Łukasz Rypicz, Piotr Karniej, Izabela Witczak, Anna Kołcz

**Affiliations:** ^1^ Department of Health Care Economics and Quality Wroclaw Medical University Wroclaw Poland; ^2^ Department of Organisation and Management Wroclaw Medical University Wroclaw Poland; ^3^ Laboratory of Ergonomics and Biomedical Monitoring Wroclaw Medical University Wroclaw Poland

**Keywords:** back pain, musculoskeletal pain, Nordic Musculoskeletal Questionnaire, nurses, occupational medicine, Polish observational study

## Abstract

Clinical nurses can encounter musculoskeletal pain episodes stemming from regular exposure to workplace risk factors that contribute to overloads. This study aimed to evaluate the occurrence and location of work‐related musculoskeletal pain among Polish nurses. An observational and descriptive survey study was conducted among 136 nurses working in the anesthesiology, intensive care, and surgery units. An extended version of the Nordic Musculoskeletal Questionnaire was used to investigate musculoskeletal pain. Only 8% of the participating nurses experienced no musculoskeletal pain, while 85% suffered from pain in more than one location. The lower back (67%), upper back (59%), and neck (66%) were the most common pain locations. In summary, nurses experienced multiple musculoskeletal pain episodes, occurring most frequently in the spinal region (lower and upper back and neck). It is crucial to determine the real causes of musculoskeletal pain and to take appropriate preventive measures to improve workplace ergonomics.

## 1 INTRODUCTION

Current global healthcare systems face a major challenge to meet the growing needs of healthcare for older adults, and this is particularly evident, as well as a priority, in the nursing profession (Lomazzi, Jenkins, & Borisch, 2016). It directly affects nursing resources throughout Europe. In Poland, it also relates to the need to take advantage of the professional potential of older nurses due to a shortage of younger professionals (Haczyński, Skrzypczak, & Winter, 2017).

Another problem arising from the challenging demographic and organizational circumstances created by the aging population, including nurses, is the absence of regulations ensuring that the nursing profession is adapted to the needs of older staff (e.g. modification of work rhythms, shift work, individualized scope of tasks, length of shifts, etc.). In addition, an extended range of health practices can improve service delivery, access to and use of care, and the quality of care itself (Ortiz et al., 2018).

The nursing profession, especially those assisting anesthesiologists and surgeons, is characterized by a highly dynamic and static workload (pushing, pulling, shifting, lifting, handling, standing, etc.) associated with considerable (Kułagowska, 2009). Physical strain and many musculoskeletal complaints A static workload can be characterized by static working positions, excluding any movement (Knibbe & Knibbe, 2012). Elements of static work occur during motion activities such as the manual lifting and moving of loads that include prolonged body segment positions (e.g. caring for reclining, impaired, or obese patients) (Yu et al., 2016). The above activities contribute to long‐term fatigue, reduced physical fitness, and a weakened musculoskeletal system. These weak spots are particularly exposed to mechanical loading and injuries most often manifested by muscle and joint pain, particularly in the spine (Gordon & Bloxham, 2016; Suh, Kim, Jung, Ko, & Ryu, 2019).

Black, Shah, Busch, Metcalfe, and Lim (2011) evaluated the effectiveness of a transfer, lifting, and repositioning program to reduce musculoskeletal injuries among direct healthcare workers and indicated that nurses in intensive care units (ICUs) were recognized as having the highest exposure to ergonomic occupational risk factors. In addition, they were particularly vulnerable to long working hours, daily workload, shift work, dissatisfaction with work, occupational burnout syndrome, caring for patients with comorbidities, recurrent deaths, interaction with patients' families, and insufficient income (Lemo et al., 2012).

Sezgin and Esin (2015), in their cross‐sectional study on a sample of 1515 ICU nurses, noted that musculoskeletal pain symptoms were mostly found in the legs and lower back, and nurses experienced pain episodes or discomfort during the previous month. Ovayolu, Ovayolu, Genc, and Col‐Araz (2014) revealed that 84.2% of nurses who worked in ICU wards (surgical, internal medicine, cardiology, pediatric, and reanimation) experienced lower back pain, 66.7% of which was moderately severe. Additionally, nurses untrained in spinal pain (86.5%), those who intermittently applied body mechanics during interventions (56.3%), remained standing for long periods (97.9%), carried out interventions while bending forward (95.8%), lifted patients (68.8%), changed their sheets while patients were in bed (65.6%), changed their position (83.3%) and did not use any special equipment during interventions (60.4%), experienced higher intensity spinal pain more frequently (Ovayolu et al., 2014).

Accordingly, we assumed that nurses with professional experience in the anesthesiology, intensive care, and surgery units were particularly exposed to overloading of the musculoskeletal system, predisposing spinal pain.

### 1.1 Literature review

The literature indicates that anesthesiology nurses face a high risk of spinal overload during both dynamic and static work (Krzemińska, Szetelnicka, Borodzicz, Arendarczyk, & Bąk, 2017; Yasobant & Rajkumar, 2014). Nurses are particularly exposed to movement system overloads (Yan et al., 2018). They perform most of their occupational activities from the front of the torso, with the body's center of gravity moving forward. An ineffective and non‐ergonomic body posture while performing professional activities results in musculoskeletal pain (Serranheira, Sousa‐Uva, & Sousa‐Uva, 2015).

The main cause of strain on the musculoskeletal system (Sierakowska et al., 2019) is that, while performing particular professional activities, anesthesiology and instrument nurses are required to maintain a forced body position for long periods of time and to lift and carry equipment of considerable mass. These constant and repetitive non‐ergonomic movements cause functional changes and reduced body efficiency and lead to pain and injuries that reduce performance. These functional limitations and disorders result in severe mental and physical fatigue (Habibi, Taheri, & Hasanzadeh, 2015). Physical fatigue leading to muscle weakness manifests itself in reduced mobility that may, in the long term, cause permanent loss of work ability (Koyuncu & Karcioglu, 2018; Phongamwong & Deema, 2015). Musculoskeletal dysfunction in nurses can lead to increased sick leave, resignation from the profession due to health conditions, and general deterioration of health (Amaro et al., 2018; Demou et al., 2018).

### 1.2 Study purpose

The primary purpose of this study was to evaluate the occurrence and specific location of work‐related musculoskeletal pain in a group of professionally active clinical nurses in Poland. The secondary purpose was to compare the occurrence of pain and its location related to age, seniority, and type of workplace. The findings of this study would provide empirical evidence and practical knowledge for the design of specific preventive and educational measures for clinical nurses regarding workplace ergonomics and the prevention of work‐related musculoskeletal disorders.

## 2 METHODS

### 2.1 Design and setting

This study was an observational and descriptive survey conducted from January to March 2019 in the anesthesiology, intensive care, and surgery units at the Wroclaw Medical University, Poland. This study followed the Strengthening the Reporting of Observational Studies in Epidemiology (STROBE) guidelines for reporting observational studies (von Elm et al., 2014).

### 2.2 Participants

The inclusion criteria for participants in this study were: (i) a master's degree (MNS); (ii) having the right to practice a profession and the status of registered nurse (RN); (iii) professional experience in anesthesiology, intensive care, or surgery units for at least 1 year; and (iv) consenting to voluntary participation in the study and understanding the objectives.

Based on a pilot study (unpublished), the estimated sample size was calculated using an analysis of the incidence of pain experienced by nurses and incorporating the following parameters: effect size – 0.3 (based on the pilot study, unpublished), degrees of freedom (1), alpha level of 0.05, and power of the test at 0.9. Based on these parameters, the estimated sample size was 117 participants. It was assumed that 30% of the questionnaires may be incomplete. The final sample size was 153 participants.

### 2.3 Measurement tool

The occurrence and location of work‐related musculoskeletal pain were evaluated by using the extended version of the Nordic Musculoskeletal Questionnaire (NMQ‐E) developed by Dawson, Steele, Hodges, and Stewart (2009). Reliable data regarding the onset, prevalence, and consequences of musculoskeletal pain in nine body regions—neck, shoulder, upper back, elbow, wrist/hand, lower back, hip/thigh, knee, and ankle/foot—were collected using a questionnaire that took approximately 10–15 min to complete (Baldwin et al., 2017). The NMQ‐E showed good construct validity, reliability, and excellent internal consistency with a Cronbach's alpha of 0.896. The NMQ‐E instrument to evaluate the occurrence and location of work‐related musculoskeletal pain is freely available for research use and does not require any permissions for use and/or translation.

### 2.4 Data collection

During their initial briefing at the beginning of January 2019, the NMQ‐E questionnaires were distributed to the participating nurses. Initially, the aim and the applied tools of the research study were discussed, and participants were assured that the study was both voluntary and anonymous. Once completed, the questionnaires were left in an agreed‐uon location (the nurse's office) in sealed envelopes. At the end of March 2019, the researchers collected and statistically analyzed these questionnaires. A total of 153 NMQ‐E questionnaires were distributed to the participants, of which 141 were returned (92% return rate). Five questionnaires were incomplete, leaving 136 questionnaires for the final analysis. The data related to participants' age, gender, seniority, and workplace (ward/unit) were collected using an additional questionnaire.

### 2.5 Ethical considerations

The study was conducted with the approval of the Bioethics Committee of Wroclaw Medical University (approval no. KB‐184/2019). All participants provided written informed consent following an explanation of the procedures involved. They were instructed that participation was voluntary and anonymous and that they could skip any questions they did not feel comfortable answering or withdraw from the study at any time. The study was carried out in accordance with the ethical guidelines of the Declaration of Helsinki (as revised in Brazil in 2013).

### 2.6 Statistical analysis

The data obtained during the survey were collated using Microsoft Excel. Statistical analyses were conducted using Statistica version 13.1 (TIBCO Software Inc., USA) under the license of the Wroclaw Medical University, Poland. The frequency of occurrence (percentage) for the qualitative variables, and the arithmetic means, standard deviations, and ranges of variation (extreme values) for the quantitative variables, were calculated. The data for quantitative sociodemographic and clinical variables were analyzed using Pearson's chi‐square test. The level of statistical significance for this study was set at *P* < 0.05.

## 3 RESULTS

### 3.1 Participants' general characteristics

The study group consisted of women with a mean age of 42.9 years (SD = 10.1). Additional age categories are presented in Table 1. Marital status was as follows: 69.9% were married, 26.5% were single, and 3.6% were divorced. According to their BMI, almost half of the participating nurses were of normal weight (47.6%), 33.9% overweight, and 18.5% were classified as obese. The vast majority of participants lived in urban areas (82.4%) as opposed to villages (17.6%). The educational level was as follows: bachelor's degree (0%), master's degree (98.5%), and PhD (1.5%). Regarding seniority, almost 39% of the respondents had worked in the profession for less than 10 years, 20% had worked for 10–20 years, and 42% had worked for more than 20 years. More than half of the respondents (53.6%) worked in the surgery unit, and the rest of the nurses (46.4%) worked in the anesthesiology and intensive care units (Table 1).

**TABLE 1:** Characteristics of the study group

Characteristic (variable)	Total n = 136	
Age; M ± SD (Min–Max)	42.9 ± 10.1 (24–70)	

	n	%
Female	136	100
Age group (years)		
<40	46	33.8
40–50	53	39.0
>50	37	27.2
Body mass index		
Normal	64	47.6
Overweight	45	33.9
Obese	27	18.5
Marital status		
Single	36	26.5
Married	95	69.9
Divorced	5	3.6
Residence		
Urban	112	82.4
Village	24	17.6
Education		
BNS	0	0
MNS	134	98.5
PhD	2	1.5
Seniority (years)		
<10	52	38.5
10–20	27	20.0
>20	56	41.5
Workplace (ward/unit)		
Surgery	73	53.7
Anesthesiology and ICU	63	46.3

%, percentage; BNS, bachelor of nursing; ICU, intensive care unit; M, mean; Max, maximum value; Min, minimum value; MNS, master of nursing; n, number of participants; PhD, post‐doctoral researcher; SD, standard deviation.

### 3.2 Number and location of pain episodes

Regarding the number of pain episodes, only 8% of respondents experienced no musculoskeletal pain in the nine body regions. Including any time in the past, the lower back (67%) was reported as the most frequent pain location, followed by the neck (66%), upper back (59%), shoulder 43%), ankle/foot (42%), knee (42%), wrist/hand (42%), and hips/thigh (38%).

Pain localized in the elbow (32%) (Table 2) was the least frequent. The average age at which participants started to experience pain ranged from 30 to 34 years, depending on the location of the pain. Participants reported neck pain as the most prevalent reason for hospitalization (48%). Lower back pain (almost 15%) was the most common basis for changes in duties or assignments at work. During the previous 1 or 12 months and on the day of completing the questionnaire, the study participants experienced neck or lower back pain most often, resulting in an inability to perform their normal duties, consulting a doctor, physiotherapist or other such person, taking medication, or applying for sick leave (Table 2).

**TABLE 2:** Participant musculoskeletal symptoms – results of NMQ‐E

Location															During the last 12 months have you at any time:							
	Have you ever had trouble (ache, pain or discomfort) in:		At the time of initial onset of the trouble, what was your age? (years)		Have you ever been hospitalized because of the trouble?		Have you ever had to change jobs or duties (even temporarily) because of the trouble?		Have you had trouble (ache, pain or discomfort) at anytime during the last 12 months?		Have you had trouble (ache, pain or discomfort) at anytime during the last months (4 weeks)?		Have you had trouble (ache, pain or discomfort) today?		Been prevented from doing your normal work (at home or away from home) because of trouble?		Seen a doctor, physiotherapist or other such person because of trouble?		Taken medication because of trouble?		Taken sick leave from work/studies because of trouble?	
	n	%	M	SD	n	%	n	%	n	%	n	%	n	%	n	%	n	%	n	%	n	%
Neck	90	66.2	32.9	8.8	65	47.8	12	8.8	81	59.6	80	58.8	47	34.6	41	30.1	50	36.8	45	33.1	25	18.4
Shoulder	59	43.4	33.1	11.0	14	10.3	6	4.4	55	40.4	47	34.6	25	18.4	27	19.9	31	22.8	29	21.3	19	14.0
Upper back	80	58.8	32.6	9.7	4	2.9	12	8.8	73	53.7	64	47.1	42	30.9	36	26.5	46	33.8	37	27.2	22	16.2
Elbow	44	32.4	32.7	13.7	11	8.1	4	2.9	34	25.0	37	27.2	17	12.5	20	14.7	23	16.9	24	17.6	16	11.8
Wrist/hand	57	41.9	33.5	14.0	3	2.2	8	5.9	52	38.2	45	33.1	21	15.4	29	21.3	27	19.9	27	19.9	19	14.0
Lower back	91	66.9	31.2	9.9	11	8.1	20	14.7	82	60.3	84	61.8	56	41.2	54	39.7	56	41.2	54	39.7	28	20.6
Hip/thigh	52	38.2	30.4	11.7	16	11.8	12	8.8	44	32.4	42	30.9	26	19.1	32	23.5	25	18.4	21	15.4	13	9.6
Knee	57	41.9	33.0	12.7	6	4.4	8	5.9	55	40.4	44	32.4	26	19.1	26	19.1	32	23.5	36	26.5	16	11.8
Ankle/foot	57	41.9	32.9	8.8	13	9.6	3	2.2	55	40.4	55	40.4	31	22.8	16	11.8	25	18.4	25	18.4	14	10.3

%, percentage; M, mean; n, number of participants; SD, standard deviation.

### 3.3 Location of pain episodes according to age

A comparative analysis of the occurrence of pain (at any time in the past) in the various body locations in relation to age, seniority, and workplace was performed (Table 3). Based on the results, no statistically significant variations were indicated regarding age (<40 vs 40–50 years old vs >50 years old) (Table 3), but increased pain symptoms in the wrist/hand, lower back, and ankle/foot were observed in groups working less than 10 years or more than 20 years compared to the group of nurses working 10–20 years (Table 3).

**TABLE 3:** Location of pain (“Have you ever had trouble: ache, pain or discomfort”) in different groups of studied nurses

Have you ever had trouble (ache, pain or discomfort) in:	Age group						
	<40 years old		40–50 years old		>50 years old		
	n	%	n	%	n	%	*P*‐value*
Neck	34	73.9	32	60.4	24	64.9	0.36
Shoulder	18	39.1	19	35.8	22	59.5	0.07
Upper back	31	67.4	25	47.2	24	64.9	0.09
Elbow	10	21.7	18	34.0	16	43.2	0.11
Wrist/hand	15	32.6	22	41.5	20	54.1	0.14
Lower back	32	69.6	34	64.2	25	67.6	0.86
Hip/thigh	21	45.7	15	28.3	16	43.2	0.16
Knee	19	41.3	19	35.8	19	51.4	0.34
Ankle/foot	17	37.0	21	39.6	19	51.4	0.38

Have you ever had trouble (ache, pain or discomfort) in:	Seniority						
	<10 years		10–20 years		>20 years		
	n	%	n	%	n	%	*P*‐value*
Neck	39	75.0	14	51.9	37	66.1	0.12
Shoulder	22	42.3	8	29.6	29	51.8	0.16
Upper back	32	61.5	14	51.9	34	60.7	0.68
Elbow	18	34.6	4	14.8	22	39.3	0.08
Wrist/hand	21	40.4	6	22.2	30	53.6	**0.024**
Lower back	39	75.0	13	48.1	39	69.6	**0.049**
Hip/thigh	21	40.4	9	33.3	22	39.3	0.82
Knee	23	44.2	9	33.3	25	44.6	0.58
Ankle/foot	28	53.8	6	22.2	23	41.1	**0.026**

Have you ever had trouble (ache, pain or discomfort) in:	Workplace (unit/ward)				
	Surgery		Anesthesiology and ICU		
	n	%	n	%	*P*‐value*
Neck	43	58.9	47	74.6	0.054
Shoulder	33	45.2	26	41.3	0.64
Upper back	40	54.8	40	63.5	0.30
Elbow	23	31.5	21	33.3	0.82
Wrist/hand	33	45.2	24	38.1	0.40
Lower back	42	57.5	49	77.8	**0.012**
Hip/thigh	28	38.4	24	38.1	0.98
Knee	27	37.0	30	47.6	0.21
Ankle/foot	25	34.2	32	50.8	0.051

Statistically significant values were marked with Bold (*p* < 0.05)

* χ^2^ test.

%, percentage; n, number of participants.

Nurses working in the anesthesiology and ICUs reported lower back pain significantly more often (*P* = 0.012) than nurses working in the surgery unit (Table 3).

## 4 DISCUSSION

As their specific duties require, anesthetic and surgical nurses often need to stand for a prolonged period. Static load‐bearing during their work manifests as pain in the musculoskeletal system. Musculoskeletal disorders regularly cause an inability to work, reduced quality of life, increased sick leave, and early retirement (Ellapen & Narsigan, 2014; Lunde et al., 2014). Aside from a shortage of nurses, this may also affect the healthcare system economically. Musculoskeletal disorders can cause disabilities in the workplace, which in turn contributes to the loss of productivity in the member states of the European Union. Estimates suggest that the total cost of this lost productivity may amount to approximately 2% of member states' gross domestic product (Bevan, 2015).

Nursing is a high‐risk profession incorporating a high risk of spinal overload. The causes of musculoskeletal pain among nurses are multifactorial. As mentioned above, the results of the cumulative research are worrying. The present study found that among 136 anesthesiology, intensive care, and surgical nurses, as many as 92% reported musculoskeletal pain; nearly one third of the respondents (28%) reported ailments located in seven to nine places; only 7% of the pain complaints were located in just one place. Causative factors may be poor work organization, excessive exploitation at work, non‐ergonomic working conditions, underestimation of the principles of work ergonomics or the age of the nurses (Marshall, Villeneuve, & Grenier, 2018). It should be noted that the average age of the participating nurses was 43 years, and 42% had worked in their field for longer than 20 years. These factors may influence the occurrence of musculoskeletal pain. In this study, age was not observed as a factor affecting the frequency of pain episodes. However, nurses with more than 20 years of professional experience have often suffered with pain. According to Arvidsson et al. (2016), the incidence of musculoskeletal disorders in the general population increases with age, which may be associated with increased seniority.

Tinubu, Mbada, Oyeyemi, and Fabunmi (2010) stress that musculoskeletal disorders are very common in healthcare workers, and particularly nurses, as their work involves direct contact with patients. Thereafter, nursing aids (nursing assistants) and paramedics experience the most musculoskeletal pain. Nurses specializing in anesthesiology and surgery are exposed to high static loads caused by prolonged standing (e.g. during surgical procedures, monitoring patients under anesthesia, overseeing patients' overall condition, moving patients from the operating table to a wheelchair, etc.) (Arvidsson et al., 2016). However, Maciuk, Krajewska‐Kulak, and Klimaszewska (2012) state that the main factor increasing the risk of developing musculoskeletal system ailments is the load resulting from working in forced positions, such as an inclined position. Pain is most commonly associated with the lumbar and cervical segments of the spine.

In this study, we compared anesthesiology and ICU nurses with surgical nurses because we assumed that their specificity of movement at work and work skills varied. Our study revealed that nurses working in the anesthesiology and ICUs experienced a significantly higher occurrence of lower back pain than nurses working in the surgery unit, proving that the lower spinal region is more overloaded in these specific work areas.

It was revealed that anesthesiology, intensive care, and surgical nurses experienced pain in the lower and upper back (67 and 59%, respectively) and neck (66%) stemming from prolonged standing and forced positions required in their scope of duties and the nature of their work. It should be noted that the profile of hospitalized patients has also changed over the years. At present, the proportion of older patients with reduced mobility along with overweight patients is increasing. Both of these patient groups affect the static load on the spine and joints of nurses' upper and lower extremities. The lack of cooperation between patients and nurses (inertia) while changing the body position or transferring the patient and the significant body weight of patients has a negative impact on nurses' musculoskeletal system and resultant pain.

It was established that nurses working for 10–20 years experienced fewer pain episodes compared with those working less than 10 years. Nurses with a shorter period of professional experience may lack knowledge of the ergonomic risk factors at work and underestimate their potential health consequences. Nurses with greater seniority may have adapted to or become familiar with the discomfort and pain accompanying the performance of their professional duties and have more experience in applying the principles of work safety. Furthermore, it should be noted that a higher occurrence of musculoskeletal pain in all the specified locations was observed with seniority >20 years compared to 10–20 years. By explanation, nurses working for more than 20 years complained about various musculoskeletal overloads due to their many years of hard work in this challenging profession. Our previous study revealed that 68% of nurses stated that they knew the principles of work ergonomics, but only 14% acknowledged that they always applied them, 62% sometimes applied them, and as many as 24% did not follow the principles of ergonomics at work, despite their awareness (Kołcz, Główka, Kowal, & Paprocka‐Borowicz, 2019).

In addition, a significant increase in work‐related musculoskeletal pain in the upper (wrist/hand) and lower limbs (ankle/foot) and spine (lower back) was observed in nurses working for less than 10 years or more than 20 years, compared to nurses working 10–20 years. Guan et al. (2019) found that a total of 51.6% nurses had pain and 31.7% had diagnosed ailments: 33.1% had lumbar spondylosis, 20.6% had dysmenorrhea, and 19.0% had cervical spine disease. The chronic pain episodes were generally moderate to severe, localized in the spine and lower limbs, and almost half the nurses believed that this pain affected their daily life and sleep quality. Heidari, Borujeni, Rezaei, and Kabirian Abyaneh (2019) reported that the most prevalent work‐related musculoskeletal disorders in nurses were localized in the spine (88.33%), knees (83.33%), and thighs (71%). In the under 25 versus 35–45 year age group, disorders occurred primarily in the neck (11 vs 39.1%), shoulders (8.3 vs 37.4%), and knees (19.56 vs 62.4%). No significant relationship was found between marital status, having a second job, working system, pain location, or education level and work‐related musculoskeletal disorders. Akodu and Ashalejo (2019) found that 60% of nurses had experienced musculoskeletal pain in the previous 12 months, predominantly in the lower back (43.2%), and the remaining pain episodes occurred in the knees, shoulders, and upper back (9.9% each). It is worth noting that almost half of the nurses (47.4%) reported good work ability, and 92.6% reported that their work ability was physically and psychologically demanding.

Previous studies have analyzed that there is a relationship between the location of the pain and its causes. It has been confirmed by numerous studies that the most common pain experienced by nurses is located in the lower back, upper back, and neck (Davis & Kotowski, 2015; Koohpayehzadeh, Bahrami‐Ahmadi, Kadkhodaei, Mortazavi, & Amiri, 2016; Moreira, Sato, Foltran, Silva, & Coury, 2014). These ailments are a major problem for medical staff, especially nurses, as they have a negative impact on their job satisfaction, quality of life, and mobility (Ellapen & Narsigan, 2014). Too great a workload, especially physical strain, can adversely affect the quality of their work.

Nowadays, when talking about musculoskeletal disorders, the aspect of ergonomics in the workplace must not be overlooked. The right ergonomic solutions are increasingly being used in the healthcare system. There are devices that are designed to be user‐friendly with the aim of safeguarding users' health. Considering nurses' working conditions, it is necessary to pay attention to the ergonomics of the positions in which they perform their duties. Nurses can improve their ergonomics if their employers provide them with suitable equipment such as adjustable stools and patient beds, mechanical lifts to help move patients, etc. (Freitag et al., 2014; Olds, Aiken, Cimiotti, & Lake, 2017). For surgical nurses, an interesting observation involves their work at the operating table. Usually, operating teams are composed of people of different heights. Assuming that the surgeon is the tallest person on the team, the correct setting of the operating table in relation to the height of the surgeon should be noted beforehand so that other members of the team (in this case, the shorter ones) can be equipped with adjustable stools to eliminate height differences that cause strain.

### 4.1 Limitations

This study has several potential limitations. First, important health and personal descriptive information such as the level of regular physical activity, performing daily housework, having children, having a second job or smoking cigarettes was not analyzed in our study as potential factors in work‐related musculoskeletal pain; however, these aspects should be taken into consideration in further studies. Second, 5% of the NMQ‐E questionnaires were returned incomplete. One solution would be to collect data personally (through interviews), although health data may be too personal for some people who may then refuse to take part in the project. Third, in the occupational group of nurses, it is not possible to extend the findings and perform gender‐related analyses; the participants' demographics are the result of the feminization of this occupation.

## 5 CONCLUSION

Nurses experienced numerous work‐related musculoskeletal pain episodes, which may cause absenteeism due to illness or resignation from the profession. Nurses reported that the most common pain location was the spinal region (lower and upper back and neck). It was demonstrated that most nurses had one or more painful conditions and/or work‐related musculoskeletal disorders. Nurses with medium seniority encountered less pain compared with those with less or greater professional experience. Nurses working in the anesthesiology and ICUs faced a significantly higher occurrence of lower back pain than nurses working in the surgery unit.

## 6 RELEVANCE FOR CLINICAL PRACTICE

Given the shortage of nurses, an increase in sick leave would reduce the quality of service to patients and jeopardize their safety. It is crucial to determine the real causes of musculoskeletal pain and to take appropriate preventive action to improve workplace ergonomics and manage nurses' physical activity. These actions may directly translate into improved health for nurses. An analysis of pain in nurses' musculoskeletal systems can be used to create a prophylactic program aimed at activating movement and improving general physical health, which is vital when working with high static loads. Nurses should be trained to use the principles of ergonomics in their daily work duties (e.g. correct lifting of a patient, changing the position of a prone patient, etc.).

## CONFLICT OF INTEREST

No conflict of interest has been declared by the authors.

## AUTHOR CONTRIBUTIONS

Study design: Ł.R., P.K, and A.K.

Data collection: ŁR. and I.W.

Data analysis: Ł.R. and A.K.

Manuscript writing: Ł.R., I.W., and A.K.

Manuscript revisions for important intellectual content: A.K. and P.K.
